# Diffusion kurtosis imaging evaluating epithelial–mesenchymal transition in colorectal carcinoma xenografts model: a preliminary study

**DOI:** 10.1038/s41598-017-11808-7

**Published:** 2017-09-12

**Authors:** Huanhuan Liu, Wenbin Shen, Caiyuan Zhang, Yanfen Cui, Jinning Li, Tingting Zhang, Weibo Chen, Dengbin Wang

**Affiliations:** 1 Department of Radiology, Xinhua Hospital, Shanghai Jiao Tong University School of Medicine, No. 1665 Kongjiang Road, Shanghai, 200092 China; 2 Department of Colorectal Surgery, Xinhua Hospital, Shanghai Jiao Tong University School of Medicine, No. 1665 Kongjiang Road, Shanghai, 200092 China; 3 Philips Healthcare, No.1 Building, 10, Lane 888, Tian Lin Road, Shanghai, 200233 China

## Abstract

Epithelial-mesenchymal transition (EMT) plays an important role in aggravating invasiveness and metastatic behavior of colorectal cancer (CRC). Identification of EMT is important for structuring treatment strategy, but has not yet been studied by using noninvasive imaging modality. Diffusion kurtosis imaging (DKI) is an advanced diffusion weighted model that could reflect tissue microstructural changes *in vivo*. In this study, EMT was induced in CRC cells (HCT116) by overexpressing Snail1 gene. We aimed to investigate the value of DKI in identifying EMT in CRC and decipher the correlations between DKI-derived parameters and EMT biomarker E-cadherin and cell proliferative index Ki-67 expression. Our results revealed that HCT116/Snail1 cells presented changes consistent with EMT resulting in significant increase in migration and invasion capacities. DKI could identify CRC with EMT, in which the DKI-derived parameter diffusivity was significantly lower, and kurtosis was significantly higher than those in the CRC/Control. Diffusivity was negatively and kurtosis was positively correlated with Ki-67 expression, whereas diffusivity was positively and kurtosis was negatively correlated with E-cadherin expression. Therefore, our study concluded that DKI can identify EMT in CRC xenograft tumors. EMT-contained CRC tumors with high Ki-67 and low E-cadherin expression were vulnerable to have lower diffusivity and higher kurtosis coefficients.

## Introduction

Colorectal cancer (CRC) is one of the most common malignant tumors in the world. With the improvement of surgical techniques and chemoradiotherapy, the 5-year survival rate of patients with rectal cancer has increased during the past decade^1^. However, tumor recurrence and metastasis are still major causes of death in patients with rectal cancer^2^. Epithelial-mesenchymal transition (EMT) is a series of molecular changes endowing epithelial cells with mesenchymal properties^3^, which is a critical process enables tumor cells to migrate and metastasize to distant sites^4^. According to the previous research, EMT is characterized by repression of membranous E-cadherin expression, and overexpression of Snail1, N-cadherin, and nuclear β-catenin proteins^3^. EMT has been documented as a potential biomarker for metastatic presence in several malignant tumors; meanwhile, it has been associated with chemoresistance in other tumors, including CRC^3, 5–11^. Therefore, identification of CRC with EMT is important for structuring treatment strategy and predicting the prognosis, since EMT may provide a predictive biomarker to tailor the neoadjuvant therapies and a novel therapeutic target to allow for improved response to neoadjuvant therapies^11^.

Currently, magnetic resonance imaging (MRI) has been widely implemented in diagnosing and staging CRC for its excellence in depiction of soft tissue by means of various anatomical and functional imaging sequences. Recently, an advanced diffusion-weighted imaging (DWI) model, diffusion kurtosis imaging (DKI) has been increasingly implemented for providing more precise information of tissue characteristics than the standard DWI does. Compared with the standard DWI assuming Gaussian behavior of water diffusion, DKI could quantify non-Gaussian behavior of water diffusion, which is closer to the movement and distribution of water molecules within biologic tissues^12^. DKI could not only provide a corrected apparent diffusion coefficient (ADC) but also reflect the deviation of tissue diffusion from a Gaussian distribution^13^.

DKI has demonstrated its capacities in different clinical applications, such as diagnosis of Parkinson’s disease, prediction of neoadjuvant chemotherapy response in nasopharyngeal carcinoma, differentiation of breast lesions, treatment assessment in hepatocellular carcinoma, and so on^12, 14–16^. However, to our best knowledge, no study has been performed on the application of DKI in identifying CRC with EMT. Therefore, the aims of our study were to investigate the feasibility of DKI in identifying CRC with EMT, and to evaluate associations between DKI-derived parameters and EMT biomarker E-cadherin expression and cell proliferative index Ki-67 expression.

## Results

### HCT116 overexpressing Snail1 displayed EMT-related phenotypes

**Figure 1:**
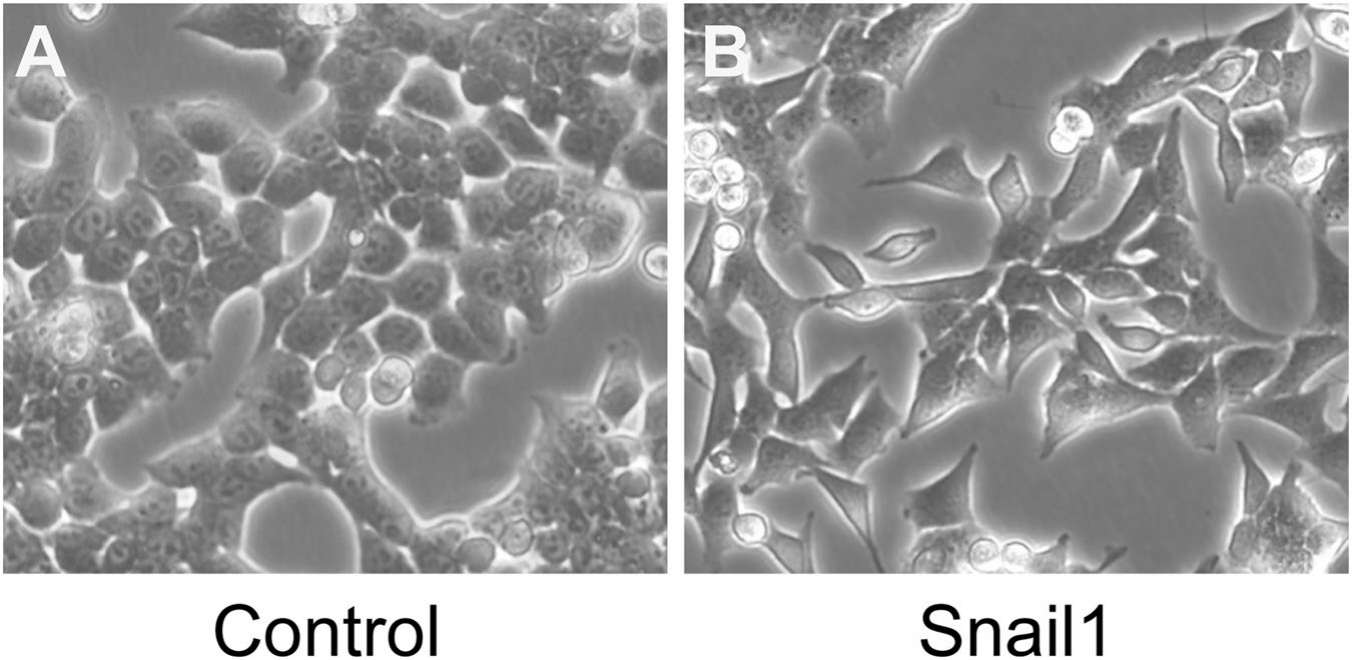
Morphologic anylysis of HCT116/Control and HCT116/Snail1 cells. The HCT116/Snail1 cells overexpressing Snail1 (B, 200× ) showed morphologic changes consistent with EMT including spindle shape with loss of cell polarity, increased intercellular separation, and increased formation of pseudopodia but not in HCT116/Control cells (A, 200× ).

The light-microscope revealed a marked altered cellular morphology for HCT116/Snail1 cells compared with the parental cells. Phenotypic changes included loss of cell polarity showing spindle shape, increased intercellular separation, and increased pseudopodia formation (Fig. 1). These changes were typical of mesenchymal cells.

### EMT-related biomarkers expression in HCT116 cell line

**Figure 2:**
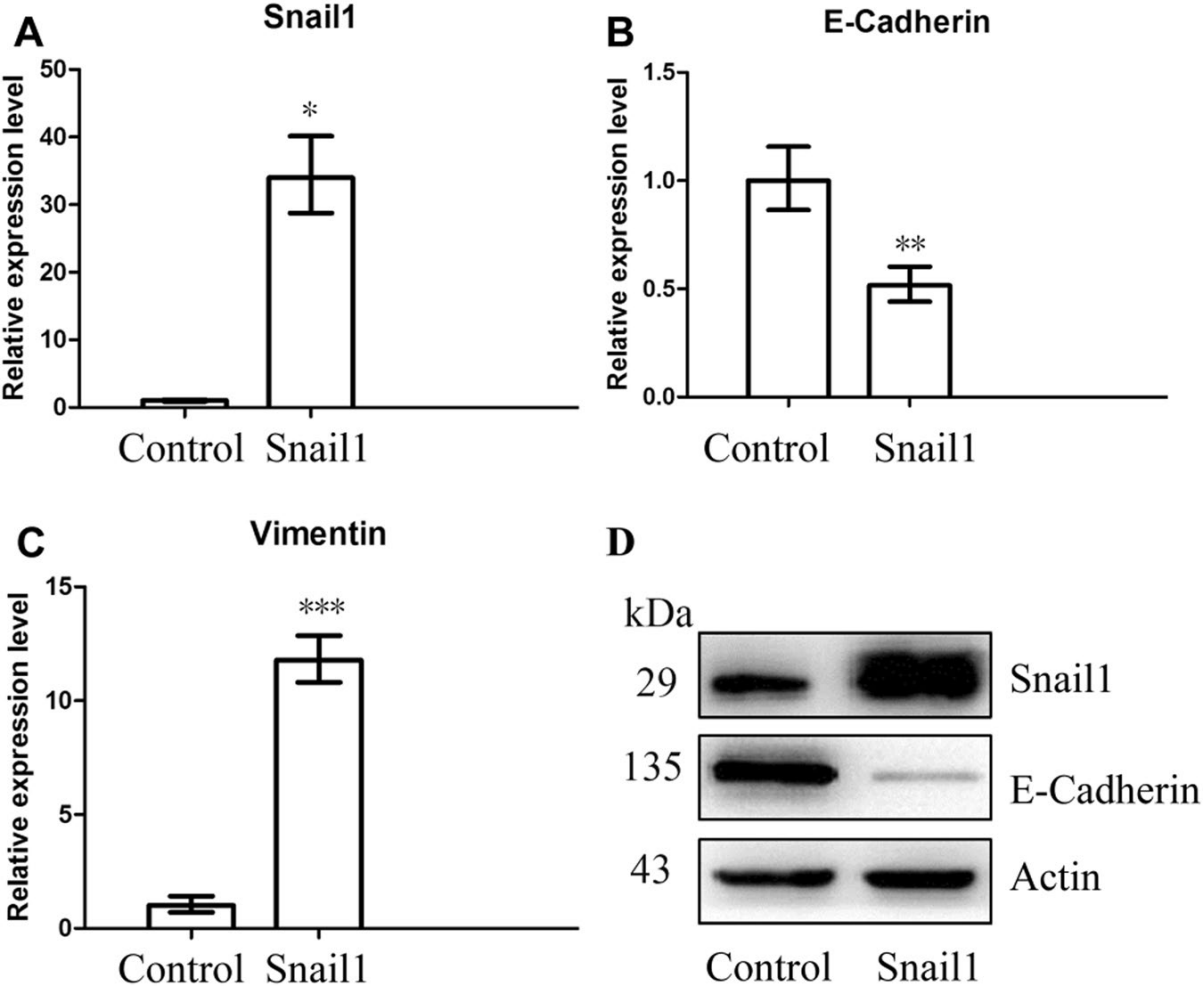
RT-PCR and Western blot analysis of HCT116/Control and HCT116/Snail1 cells. RT-PCR analysis showed that Snail1-mRNA expression (**A**) was significantly higher (*P* = 0.001), E-cadherin-mRNA expression (**B**) was significantly lower (*P* = 0.007), and vimentin-mRNA expression (**C**) was significantly higher (*P* < 0.001) in HCT116/Snail1 cells than those in the HCT116/Control. Expression was normalized to GAPDH. Western blot analysis showed that Snail1 protein expression was higher and E-cadherin protein expression was lower in HCT116/Snail1 cells as compared with the HCT116/Control (**D**). β-actin was used as loading control. kDa, molecular weight. The images of bands were cropped from different parts of the same gel. The uncropped images of bands were presented in Supplementary Figure S1.

Compared with the parental cells, quantitative real-time polymerase chain reaction (RT-PCR) analysis revealed that HCT116/Snail1 cells demonstrated a 33.9-fold increase in Snail1-mRNA expression (*P* = 0.001) (Fig. 2A), 50% decrease in E-cadherin-mRNA expression (*P* = 0.007) (Fig. 2B), and an 11.8-fold increase in vimentin-mRNA expression (*P* < 0.001) (Fig. 2C).

Western blot analysis demonstrated that Snail1 protein expression was up-regulated and E-cadherin protein expression was down-regulated in HCT116/Snail1 cells compared with HCT116/Control cells (Fig. 2D).

### HCT116 overexpressing Snail1 increased migratory and invasive capacity

**Figure 3:**
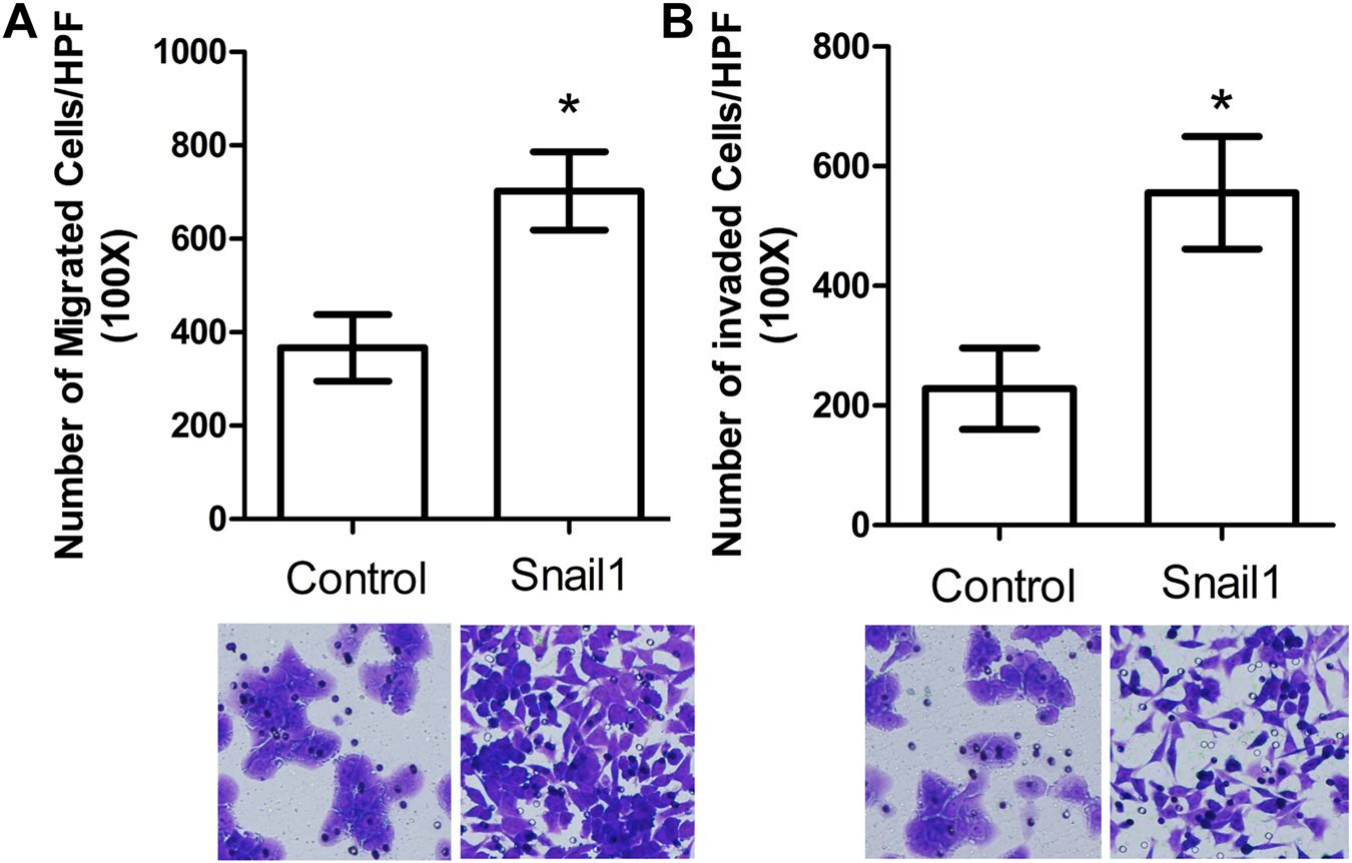
Migration and invasion results of HCT116/Control and HCT116/Snail1 cells. HCT116/Snail1 cells increased number of migrating cells (A, *P* < 0.001) and invading cells (B, *P* < 0.001) per microscope field compared with HCT116/Control cells.

In the migration and Matrigel invasion assays, HCT116/Snail1 cells demonstrated a 1.9-fold increase in migration (*P* < 0.001), and a 2.4-fold increase in invasion (*P* < 0.001) compared with HCT116/Control cells (Fig. 3).

### Summary of kurtosis metric

The CRC/Control and CRC/EMT xenograft tumors models were successfully established among 20 female nude mice. The mean tumor volumes of CRC/Control and CRC/EMT tumors were 485.64 ± 74.05 mm^3^ (range, 415.15-653.46 mm^3^) and 469.59 ± 44.29 mm^3^ (range, 418.97–527.59 mm^3^) (*P* = 0.558). The median time interval between the tumor implantation and MR imaging was 14 days (range, 12–16 days) in the CRC/Control group and 10 days (range, 10–14 days) in the CRC/EMT groups, which showed significant different (*P* < 0.001). R^2^ value was calculated to assess the goodness-of-fit for kurtosis models. The meanR^2^ value for kurtosis fitting of 0.999 ± 0.001 (range, 0.999–1.000). The summary of ADC, diffusivity, and kurtosis values for CRC/Control and CRC/EMT tumors was provided in Table 1. Compared with the CRC/Control tumors, ADC and diffusivity values were significantly lower (*P* = 0.007 vs. *P* = 0.002), and kurtosis values were significantly higher (*P* < 0.001) in the CRC/EMT tumors.

**Table 1:** ADC, Diffusivity, and Kurtosis of CRC/Control and CRC/EMT tumors.

Parameters	CRC/Control	CRC/EMT	*P* value
ADC^a^	0.46 ± 0.05	0.39 ± 0.04	0.007
Diffusivity^a^	0.54 ± 0.06	0.46 ± 0.02	0.002
Kurtosis	1.22 ± 0.14	1.53 ± 0.13	<0.001

Data were shown as mean values ± standard deviation.

CRC: colorectal carcinoma, EMT: epithelial-mesenchymal transition

^a^ *10^−3^mm^2^/s.

The areas under receiver operating characteristic curves (ROC) for ADC, diffusivity and kurtosis were 0.82, 0.90 and 0.95, respectively. It seems that there could be a trend where diffusivity and kurtosis have higher areas under ROC curves (AUCs) than that of ADC, though the differences among AUCs were not statistically significant (ADC vs. diffusivity, *P* = 0.421; ADC vs. kurtosis, *P* = 0.218; diffusivity vs. kurtosis, *P* = 0.664). With a threshold of 0.39 × 10^−3^ mm^2^/s for ADC, 0.48 × 10^−3^ mm^2^/s for diffusivity and 1.34 for kurtosis, diffusivity and kurtosis showed potentially higher sensitivity (Table 2).

**Table 2:** Receiver Operating Characteristic Curve Analysis of ADC, Diffusivity, and Kurtosis in distinguishing CRC/Control and CRC/EMT tumors.

Parameters	AUCs	Threshold	Sensitivity	Specificity	*P* value
ADC	0.82(0.59–0.95)	≤0.39^a^	70(34.8–93.3)	90(55.5–99.7)	0.0009
Diffusivity	0.90(0.68–0.99)	≤0.48^a^	100(69.2–100)	90(55.5–99.7)	0.0001
Kurtosis	0.95(0.75–0.99)	>1.34	100(69.2–100)	80(44.4–97.5)	<0.0001

CRC: colorectal carcinoma, EMT: epithelial-mesenchymal transition.

AUCs: areas under receiver operating characteristic curves.

Data were shown as percentage except the thresholds. Data in parentheses are 95% confidence interval.

^a^*10^−3^mm^2^/s.

For intra-observer agreement of observer 1 and observer 2, the intraclass correlation coefficients (ICCs) were 0.938 (95% confidence interval (CI), 0.869–0.973) and 0.948 (95% CI, 0.891–0.978) for ADC, 0.977 (95% CI, 0.951–0.990) and 0.975 (95% CI, 0.948–0.990) for diffusivity, and 0.970 (95% CI, 0.936–0.987) and 0.961 (95% CI, 0.919–0.983) for kurtosis, respectively. The inter-observer agreement was excellent in measurements for ADC, diffusivity, and kurtosis parameters with interclass correlation coefficients (ICCs) of 0.984 (95% CI, 0.960–0.994), 0.969 (95% CI, 0.921–0.988), and 0.877 (95% CI, 0.690–0.951), respectively.

### Histological assessment and correlations with ADC and DKI-derived parameters

**Figure 4:**
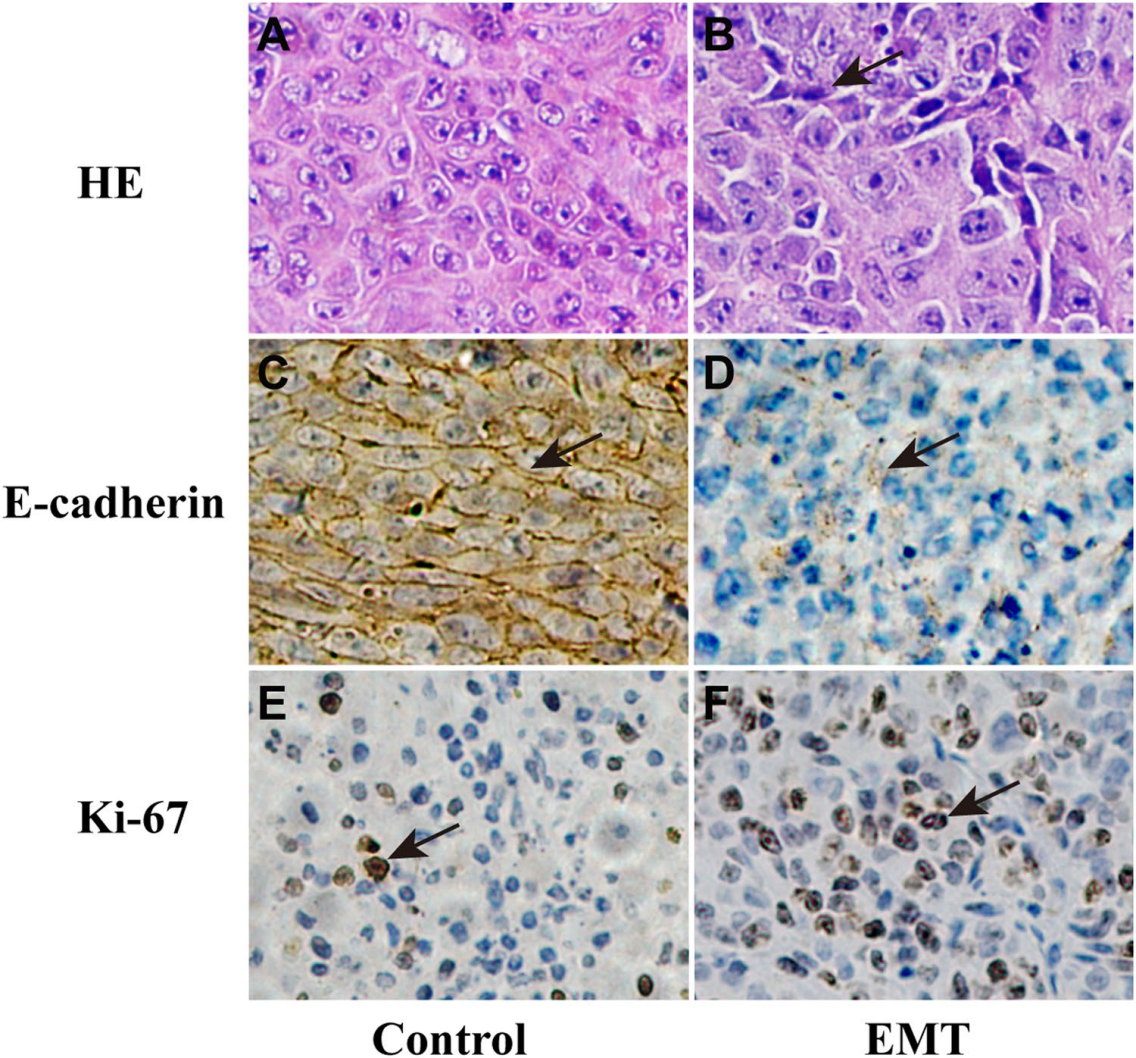
Hematoxylin and eosin (HE) and immunohistochemical analysis of representative CRC/Control and CRC/EMT tumors. The left and right columns were representative images of HE, E-cadherin, and Ki-67 staining for the CRC/Control and CRC/EMT tumors. HE staining showed that CRC/EMT cells were more heterogenenous with spindle-shaped alteration (**B**, 200× , arrow) compared with the CRC/Control (**A**, 200×). Immunohistochemical stainings showed that E-cadherin expression (**D**, 200× , arrow) was repressed, and positive Ki-67 expression (**F**, 200×, arrow) was higher in the CRC/EMT group as compared with the CRC/Control (**C**, 200× , arrow; **E**, 200×, arrow)

The mean cell count of CRC/EMT tumors was 700 ± 43 (range, 650–774), which was significantly higher than that of 488 ± 42 (range, 428–553) in CRC/Control tumors. Both ADC and diffusivity showed negative correlation with tumor cellularity (r = −0.47, *P* = 0.036 vs. r = −0.56, *P* = 0.010). Kurtosis demonstrated positive correlation with tumor cellularity (r = 0.74, *P* < 0.001). Compared with CRC/Control tumors, the CRC cells showed poorer differentiation and more heterogeneity with spindle-shaped alternation (Fig. 4A and B), down-regulation of E-cadherin expression (Fig. 4C and D), and higher Ki-67 expression in CRC/EMT tumors (Fig. 4E and F). Correlations between the diffusion parameters and immunohistochemical indexes were shown in Table 3. There was no significant correlation between ADC and E-cadherin expression (*P* = 0.068). Diffusivity was negatively correlated with Ki-67 expression (*P* = 0.007) and positively correlated with E-cadherin expression (*P* = 0.023). Kurtosis was significantly positively correlated with Ki-67 expression (*P* < 0.001) while it was significantly negatively correlated with E-cadherin expression (*P* = 0.006).

**Table 3:** Correlations between histologic features and diffusion parameters in CRC/Control and CRC/EMT tumors.

Parameters	Ki-67		E-cadherin	
	r	*P* value	r	*P* value
ADC	−0.56	0.011	0.42	0.068
Diffusivity	−0.59	0.007	0.51	0.023
Kurtosis	0.73	<0.001	−0.59	0.006

## Discussion

In the present study, EMT model was induced in HCT116 CRC cell line by overexpressing the EMT regulator Snail1. The EMT model is a well-established model in the previous studies^17, 18^, where EMT model can be induced by transforming growth factor β (TGF-β) or EMT regulators overexpression. Several transcription factors including Snail1, ZEB1, Twist, and so on have been implicated in the control of EMT, Snail1 has been reported as a central mediator of EMT among the factors and plays a role in aggravating the invasiveness and metastatic behavior of malignant neoplasms^4, 19–22^. In our study, we chose the EMT model induced by Snail1 overexpression for our further experiment and the EMT model was successfully reproduced in HCT116 CRC cell line. The mesenchymal phenotype characteristics in HCT116/Snail1 cell line were validated by morphologic observation, RT-PCR, and Western blot analysis, including loss of cell polarity, up-regulation of Snail1-mRNA and vimentin-mRNA, down-regulation of E-cadherin-mRNA and the corresponding proteins. Our results of migration and invasion assays also validated the increased migratory and invasive capacity in HCT116/Snail1 cells. More migrating and invading cells were present in HCT116/Snail1 group compared with those in HCT116/Control group. We implemented the model to decipher whether the DKI parameters were correlated with the EMT.

CRC/Control and CRC/EMT xenograft models were successfully established in our preliminary study. We chose only one time point for MR imaging when the tumor volume was about 500 mm^3^. The tumor size was similar to that in the study by Liu *et al*.^23^. We tried our best to obtain images with high quality, assess the microstructural heterogeneity of solid components in the tumor, and avoid the heterogeneity changes caused by the cystic structures when the tumor grew. Therefore, our preliminary study chose an appropriate tumor size for MR imaging instead of various sizes. In the present study, a small amount of xenograft tumors showed a little visible cystic structure. In case of tumors with obvious cystic degeneration, ROIs should be placed in the tumors to avoid the cystic areas with a reference to T2W image.

Our study revealed that DKI-derived parameters could be utilized to discriminate xenograft tumors with EMT from the CRC/Control ones. Diffusivity values were significantly lower and kurtosis values were significantly higher for tumors with EMT than those for the CRC/Control. Compared with the AUC of ADC in identifying CRC with EMT, AUCs of diffusivity and kurtosis were potential higher, implying the possible trend towards a higher diagnostic efficiency with diffusivity and kurtosis, though there were no significant differences among the AUCs values. The relatively small sample size may be partly responsible for the above conditions. Our results were similar to those in the previous studies by Zhu *et al*. and Rosenkrantz *et al*.^24, 25^, which reported that the AUCs values of ADC, diffusivity, and kurtosis showed no significant differences in differentiating high- from low-grade rectal adenocarcinomas or distinguishing prostate cancerous tissue from benign peripheral zone, respectively. However, some other clinical studies reported that DKI model outperformed conventional monoexponential ADC in discriminating rectal cancer with distant metastasis from those without distant metastasis, and malignancy from benignity of breast^16, 26^, as DKI could not only provide a corrected ADC but also reflect the deviation of tissue diffusion from a Gaussian distribution.

Several factors contributed to the improved performance with DKI model compared with the standard monoexponential one. First, in contrast to the Gaussian diffusion assumption for conventional diffusion imaging, DKI model quantifies the non-Gaussian water motion, which is closer to the diffusion behavior in tissues *in vivo*. Thereby, assessing the tissue microstructural environments with DKI model is more accurate and sensitive compared with the standard monoexponential one. Second, the increased cellularity, larger nuclear/cytoplasmic ratio, and less extracellular space always lead to more barriers within tumor tissues and higher restriction of water diffusion, where the environments were more heterogenous^27^. DKI could reflect the tissue microstructural heterogeneity. Third, Snail1 could induce stemness and cell dedifferentiation in CRC. CRC cells that had undergone the EMT due to overexpression of Snail1 had higher self-renewal ability and poorer differentiation, resulting in enhanced malignancy, tumorigenicity, and heterogeneity *in vivo* ^22^. In our study, the tumor cellularity was significantly higher in CRC/EMT tumors than those in CRC/Control ones, indicating the stimulation of proliferation in CRC/EMT cells. Furthermore, poorer differentiation and more heterogeneity were also unveiled in the CRC cells of the CRC/EMT group. These characteristics could be better reflected via DKI model by quantifying the non-Gaussian water motion and excess kurtosis in the tumors.

As for the correlations between DKI-derived parameters and immunohistochemical indexes, our study revealed that diffusivity and kurtosis were significantly correlated with Ki-67 expression, while Ki-67 expression was significantly higher in CRC/EMT tumors than that in CRC/Control ones. Ki-67 is expressed in proliferating cells from G1 to M-phase of the cell cycle, reflecting the growth fraction of a tumor^28^. Previous studies have shown the predictive role of Ki-67 in a range of malignancies, including breast cancer, gastrointestinal neuroendocrine tumors, CRC, and so on^28–30^. The higher proliferative characteristic of HCT116/Snail1 cell line could be addressed by the increased cellularity and the structural complexity due to the vascular hyperplasia^16^, which could be reflected by DKI parameters such as diffusivity and kurtosis. Our results were similar to those in the study by Jiang *et al*.^31^. Besides the cell proliferation index Ki-67, the EMT biomarker E-cadherin was also demonstrated to be significantly correlated with poor prognosis^32^. Our study visualized that diffusivity was positively and kurtosis was negatively correlated with E-cadherin expression. Therefore, DKI could be a non-invasive choice for evaluating E-cadherin expression and anti-EMT treatment assessment.

Recently, machine learning-based approaches including radiomics, radiogenomics, and so on, have drawn more and more attention and demonstrated the potential feasibility in lesion detection and classification, including but are not limited to prostate cancer, bladder cancer, glioblastoma, and non-small cell lung cancer (NSCLC)^18, 33–35^. Radiogenomics referring to the combination of imaging and gene expression is reported to have the potential to give insight into tumor biology. Heiland *et al*.^35^ reported that DWI-derived parameter fractional anisotropy (FA) was strongly associated with EMT pathway activation by network analysis with diffusion data. Yamamoto *et al*.^18^ found that integrative radiogenomic analysis showed an association between increased normalized ^18^F fluoro-2-deoxyglucose positron emission tomography (PET) high normalized maximum standardized uptake value (SUVmax), outcome, and EMT in NSCLC. Our study demonstrated that DKI could help to identify the CRC with EMT. Compared to the ^18^F–FDG PET imaging, DKI is a non-invasive imaging technique without any radiation, which enables higher specificity in lesion detection and characterization. Furthermore, MRI is more widely used in clinical context in the world. In our future investigations, radiogenomic analysis will be a promising approach to assess the associations between CRC with lower diffusivity and higher kurtosis and EMT phenotype or the molecular mechanisms.

Our study has several limitations. First, the sample size was relatively small. Further experiments with more animals and other CRC cell lines are still warranted to validate the diagnostic performance of DKI-derived parameters in identifying CRC with EMT. Second, although our results from animal models showed potential value, the study on translation of DKI into clinical application should be further warranted. Third, histogram analysis or whole-volume measurements were reported to provide more information related to the tumor structure and heterogeneity^26, 36^. Further investigations on the value of the measurement methods would be performed in the future. Finally, trace-weighted images were employed to estimate DKI maps in our study. Although we performed the procedure after referring to the previous studies, which showed the feasibility of DKI in body applications^16, 24, 25^. However, a recent study by Giannelli M and ToschiN^37^ reported that trace-weighted images to estimate the average of diffusional kurtosis values along the main orthogonal directions (K) can introduce a substantial error, and the absolute percentage error in K can range up to 60%. In that study, it is recommended that the best-possible approximation for mean diffusional kurtosis can be obtained by separately fitting the DKI model along each diffusion weighting direction {x, y, z} prior to averaging, given that the full kurtosis tensor cannot be estimated. We will improve our procedure in future investigation.

In conclusion, DKI could help to identify CRC xenograft tumors with EMT. Furthermore, diffusivity and kurtosis were significantly correlated with Ki-67 and E-cadherin expression, which may serve as a non-invasive approach in the evaluation of chemotherapeutic effects or anti-EMT therapy.

## Methods

### Cell lines and culture conditions, animals, and reagents

The human CRC cell line HCT116 was obtained from Chinese Academy of Sciences Committee Type Culture Collection cell bank (China). HCT116 was cultured in McCoy’s 5 A medium with 10% fetal bovine serum (FBS), 100 U/mL penicillin-streptomycin, 2 mM L-glutamine, and 1% nonessential amino acids (Life Technologies, Grand Island, NY) at 37 °C and 5% CO_2_. A total of 20 female BALB/c nude mice (5 weeks old, 18–20 g) were purchased from Shanghai Experimental Animal Center (Shanghai, China) and maintained in a specific pathogen-free environment. All the experimental protocols were approved by the Ethics Committee of Xinhua Hospital Affiliated to Shanghai Jiao Tong University School of Medicine. All methods were carried out in strict accordance with the relevant guidelines and regulations of the National Institutes of Health for the Care and Use Committee of Laboratory Animals.

Primary antibodies for Western blot and immunohistochemical staining were as follows: rabbit anti-Snail, rabbit anti-E-cadherin (Cell Signaling Techonology, Danvers, MA), mouse anti-β-actin (Beyotime, China), rabbit anti-Ki-67 (Cell Signaling Techonology, Danvers, MA). Secondary antibodies were HRP-label goat anti-rabbit IgG (Bioworld Technology, USA) and goat anti-mouse IgG (Beyotime, China).

### Snail1 stable cell line

The full length of Snail1 CDS region was inserted from BamHI to EcorI sites in the pBABE-puro vector. For virus package, GAG, VSVG were cotransfected into 293 T cells with pBABE-puro or pBABE-Snail1-puro by Polyethylenimine linear (PEI, polysciences). After 48 to 72 hours, virus particles were harvested. Cell lines were infected with retrovirus overnight with 1ug/ml polybrene (Sigma, USA) before replacing with fresh medium. Then, HCT116 cells were selected by using puromycin 24 hours after transfection for 1 week.

### Morphologic analysis

Cells were grown to 70% confluence in McCoy’ 5 A modified medium with 10% FBS and visualized at ×200 magnification with a light microscope (Leica DMI3000B, Germany). The morphologic characteristics of Snail1-overexpressing HCT116 cells (HCT116/Snail1) and parental cells (HCT116/Control) were compared.

### RT-PCR analysis

Total RNA was extracted from HCT116/Snail1 and HCT116/Control cells by using Trizol (TaKaRa), and quantity was determined using a NanoDrop ND-1100 (NanoDrop Technologies). Complementary DNA was synthesized with PrimeScript RT-PCR Kit (TaKaRa). RT-PCR was performed using SYBR Premix ExTaq (TaKaRa) on ABI 7500 RT-PCR system (Applied Biosystems). GAPDH was used as an internal control for normalization. Primers used to measure the expression of Snail1, E-cadherin, vimentin, and GAPDH were provided in in Supplementary Table S1.

Procedures for RT-PCR were: initial denaturation at 95 °C for 1 min, followed by 40 cycles which involved heating to 95 °C for 5 s and then 64 °C for 35 s. The relative gene expression levels were calculated using 2^−ΔΔCt^ analysis method.

### Western blot analysis

Cells were suspended in enhanced radioimmunoprecipitation assay (RIPA) protein lysis buffer (Beyotime, China) containing 1% dilution of the Phenylmethanesulfonyl fluoride (PMSF) (Beyotime, China). Protein concentration was quantified using a microplate reader (Bio-TEK, USA) with the enhanced BCA Protein Assay kit (Beyotime, China). Equal amounts (20 µg) of total protein was resolved SDS-PAGE (10% polyacrylamide gel) and transferred to a 0.45 µm polyvinylidene difluorade (PVDF) membrane (Millipore, USA). After blocking the membrane in 5% non-fat milk in Tris-buffered saline (10 mM Tris, 150 mM NaCl) containing 0.1% Tween-20 (TBS-T) for 2 hour, the membrane was incubated with primary antibodies (1:1000 dilution) at 4 °C overnight. After washing thrice in TBS-T for 10 min each time, the membrane was incubated with labeled secondary antibody (1:6000 dilution) at room temperature for 1 hour. The proteins of interest were detected using the Immobilon Western Chemilum HRP Substrate (Millipore, USA) and image was acquired using GelDoc XR System (Bio-rad, USA).

### Migration and invasion assays

HCT116/Snail1 and HCT116/Control cells (5 × 10^4^/well) were seeded into the upper chamber of a Transwell insert pre-coated with 5 µg/mL fibronectin for migration or a BD Matrigel invasion chamber for invasion in serum-free medium on each 8.0-µm pore size membrane insert in 24-well plates (BD Biosciences, San Jose, CA). McCoy’ 5 A medium containing 10% FBS was added to the lower chamber as chemoattractants. After a 48-hours incubation at 37 °C with 5% CO_2_, non-migratory cells were scraped from the membrane of the top compartment, and the migratory cells remaining on the lower surface of the insert were fixed and stained using the Crystal Violet Staining Solution (Beyotime, China). The number of cells that migrated was quantified as the average number of cells found in five random microscope fields at ×100 magnification (Olympus, Tokyo, Japan).

### Animal experiments and MR imaging

A total of 20 female nude mice were randomly divided into two groups (n = 10 per group). CRC/Control and CRC/EMT xenograft tumors were generated by injecting 5 × 10^6^ HCT116/Control and HCT116/Snail1cells in 0.2 mL serum-free media subcutaneously into the right hind flank of mouse in each group. Tumor size was measured every other day by caliper since one week after implantation. Tumor volume was calculated by using the following formula: V = 1/2 (length × width × width). When the tumor size was about 500 mm^3^, MR imaging was performed.

MR imaging was performed using a 3.0-T MR system (Ingenia, Philips, Medical System, Best, Netherlands) with an eight-channel receiver coil of 5.0 cm inner diameter (Chenguang Medical Technologies Co., Shanghai, China). Transverse T2-weighted (T2W) fast spin echo imaging and a single-shot spin-echo echo-planar DKI sequence were performed for all mice. The parameters for T2W imaging were as follows: repetition time/echo time (TR/TE) 6000/68 ms, 8–12 slices, slice thickness 1 mm, no interslice gap, field of view (FOV) 50 × 50 mm^2^, a matrix of 144 × 136. The b-values of DKI were 0,500,1000,1500 and 2000 sec/mm^2^ and the other parameters for DKI: TR/TE 3000/59 ms, 4–6 slices, slice thickness 2 mm, no interslice gap, FOV 50 × 50 mm^2^, matrix 64 × 64, parallel imaging factor 3, receiver bandwidth 1047.9 Hz, 6 signal averages. DKI was performed by using three orthogonal motion-probing gradient directions, which were geometrically averaged to generate isotropic diffusion kurtosis, or trace-weighted images.

### Image analysis

**Figure 5:**
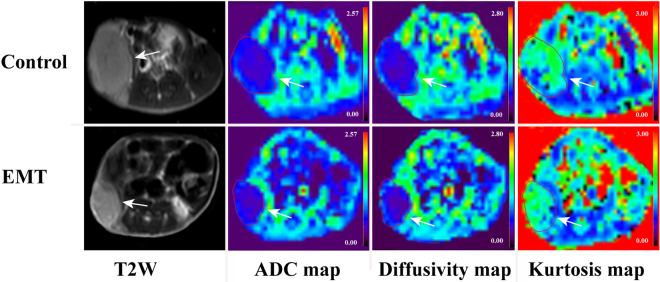
Representative MR images of CRC xenograft tumors in CRC/Control and CRC/ EMT groups. The upper and lower rows were axial T2W, ADC, Diffusivity, and Kurtosis color maps in CRC/Control tumor (arrow) and CRC/ EMT tumor (arrow) in the right hind flank, respectively. The ADC and diffusivity coefficients calculated from lesion ROI of CRC/Control tumor on the corresponding maps were (0.480 ± 0.056) × 10^−3^ mm^2^/s and (0.660 ± 0.032) × 10^−3^ mm^2^/s, respectively, which were higher than those of (0.383 ± 0.043) × 10^−3^ mm^2^/s and (0.447 ± 0.014) × 10^−3^ mm^2^/s in the CRC/EMT tumor. Kurtosis coefficient of 1.477 ± 0.061 in the CRC/Control tumor was lower than that of 1.627 ± 0.050 in the CRC/EMT tumor.

The DKI data were postprocessed by using DWI-Tool software developed by Philips (IDL 6.3, ITT Visual Information Solutions, Boulder, CO. USA)^38^. Diffusivity and kurtosis maps were calculated with the software by performing a voxel-by-voxel fitting of DKI data on the basis of robust nonlinear least-squares curve fittings based on the Levenberg-Marquardt algorithm using the equation: S = S_0_·exp(−b·D + b^2^·D^2^·K/6), in which S is DWI signal intensity at a particular b value, S_0_ is the signal intensity when b value is 0 sec/mm^2^, b represents b-value, diffusivity represents the corrected ADC accounting for non-Gaussian diffusion behavior and kurtosis represents excess kurtosis^16^. Kurtosis is a unitless parameter with 0 representing perfectly Gaussian diffusion and larger kurtosis representing greater deviation from a Gaussian fitting. The ADC map was calculated using the standard monoexponential fitting with the equation S = S_0_·exp (−b·ADC) (Fig. 5).

Two radiologists (C.Y.Z and J.N.L, with 5 and 3 years of experience in abdominal imaging, respectively), who were blinded to histopathological results, independently measured ADC, diffusivity and kurtosis values on the corresponding maps. Regions of interest (ROIs, range 57–214 pixels) were placed on the center slice of each tumor with a reference to T2W image to avoid the hemorrhagic, cystic, and necrotic areas. Each observer performed two measurements. ICCs computed from observer 1’s or observer 2’s two measurements and ICCs computed from observer 1’s first measurements and observer 2’s first measurements were used to evaluate intra- and inter-observer agreement for ADC, diffusivity, and kurtosis measurements. The mean ADC, diffusivity, and kurtosis values were calculated as an average of four measurements from observer 1 and observer 2.

### Histologic analysis

Following MRI experiments, all mice were sacrificed by cervical dislocation with deep anesthesia by means of intraperitoneal injection with pentobarbital sodium. Then, formaldehyde-fixed, paraffin-embedded tissue blocks were prepared from xenograft tissues and cut into serial sections (4 µm) for hematoxylin and eosin (HE) staining. Tumor cellularity was obtained by identifying three areas with the same size within the lesions on high-power pictures (400× magnification). The tumor cellularity was quantified as the average count from these three regions. Immunohistochemical stainings for Ki-67 and E-cadherin were performed.

### Statistical analysis

Statistical analysis was performed with SPSS 19.0 (IBM, New York, NY). Quantitative variables were expressed as means ± standard deviation.

The differences of ADC, diffusivity, kurtosis values, and the median time interval between tumor implantation and MR imaging forthe CRC/EMT and CRC/Control tumors were compared by using the Student t test and *P* < 0.05 indicated a statistically significant difference. ROC curves were generated to assess and compare ADC, diffusivity, and kurtosis in terms of their utility for identifying CRC models with EMT. For ROC analysis, sensitivity and specificity were calculated by using an optimal threshold determined as that would maximize the sum of sensitivity and specificity. The AUCs were compared by using the methods developed by DeLong *et al*.^39^. Given the three variables used for the comparison of AUCs, a *P* value of less than 0.0167 was considered indicative of a significant difference.

ICC values were considered to indicate excellent agreement if they were greater than 0.8 and substantial agreement if they were in the range of 0.6–0.79^27^. Pearson and Spearman correlation were performed to evaluate associations between diffusion parameters and tumor cellularity and immunohistochemical indexes including Ki-67 and E-cadherin expression, respectively. The correlation coefficient (r) was defined as little or no correlation (⩽0.24), fair correlation (0.25–0.49), moderate to good correlation (0.5–0.74), and good to excellent correlation (0.75–1.00)^16^.

### Data availability

All data generated or analyzed of this study are included in this published article. Raw and processed data during the current study are available from the corresponding author upon reasonable request.

## Electronic supplementary material

Supplementary Information
