# Supplementary material for: Diffusion kurtosis imaging evaluating epithelial–mesenchymal transition in colorectal carcinoma xenografts model: a preliminary study
Source: Sci Rep. 2017 Sep 12;7:11424. doi: 10.1038/s41598-017-11808-7 (PMC5595886; doi:10.1038/s41598-017-11808-7)

Diffusion kurtosis imaging evaluating epithelial–mesenchymal transition in colorectal carcinoma xenografts model: a preliminary study

Huanhuan Liu MD[1#], Wenbin Shen MD[2#], Caiyuan Zhang MD, PhD[1], Yanfen Cui MD, PhD[1], Jinning Li MD[1], Tingting Zhang MD[1],Weibo Chen PhD[3], Dengbin Wang MD, PhD[1]*

[1]Department of Radiology, Xinhua Hospital, Shanghai Jiao Tong University School of Medicine, No. 1665 Kongjiang Road, Shanghai, China, 200092

[2]Department of Colorectal Oncology and Anal Surgery, Xinhua Hospital, Shanghai Jiao Tong University School of Medicine, No. 1665 Kongjiang Road, Shanghai, China, 200092

[3] Philips Healthcare, No.1 Building, 10, Lane 888, Tian Lin Road, Shanghai, China, 200233

[#]These authors have contributed equally to this work

Corresponding Author: Dengbin Wang, Department of Radiology, Xinhua Hospital, Shanghai Jiao Tong University School of Medicine, No. 1665 Kongjiang Road, Yangpu District, Shanghai, China, 200092.

Telephone number: +86-21-25078999-7030. Fax number: +86-21-65795173.

E-mail: dbwang8@aliyun.com.

TableS1 Snail1, E-cadherin, vimentin and GAPDH primers for RT-PCR analysis

Primer Name	Forward Sequence (5′-3′)	Reverse Sequence (5′-3′)
Snail1	CTCTAGGCCCTGGCTGCTAC	TCTGAGTGGGTCTGGAGGTG
E-cadherin	TTGACGCCGAGAGCTACAC	GACCGGTGCAATCTTCAAA
vimentin	GACGCCATCAACACCGAGTT	CTTTGTCGTTGGTTAGCTGGT
GAPDH	GGGCATCTTGGGCTACAC	GGTCCAGGGTTTCTTACTCC

Figure S1 The uncropped images of bands for Snail1, E-cadherin, and β-actin proteins

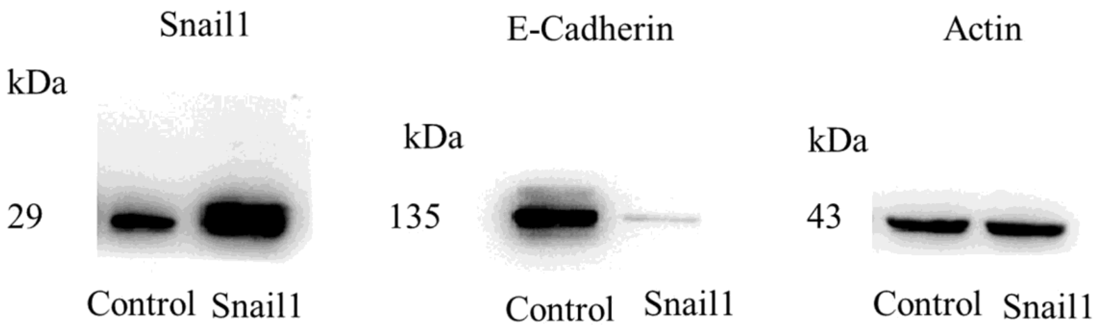

Supplement: Supplementary file 1 — Supplementary Information [file 41598_2017_11808_MOESM1_ESM.pdf]
